# Clinical Lecture on Some of the Anomalous Phenomena Complicating Cases of Typhoid and Typho-Malarial Fevers

**Published:** 1872-08-15

**Authors:** N. S. Davis

**Affiliations:** Prof. of Practical and Clinical Medicine in Chicago Medical College and Mercy Hospital


### CLINICAL LECTURE ON SOME OF THE ANOMALOUS PHENOMENA COMPLICATING CASES OF TYPHOID AND TYPHO-MALARIAL FEVERS.

BY N. S. DAVIS, PROF. OF PRACTICAL AND CLINICAL MEDICINE IN CHICAGO MEDICAL COLLEGE AND MERCY HOSPITAL.

*Gentlemen:* The patient before you is a laboring man, aged about 36 years, a native of Canada, and a resident of this city only a few months.

About one week since he called at my office for advice. His expression of countenance was dull; face moderately flushed; skin dry and warmer than natural; pulse about 90 per minute and soft; respiration natural; tongue covered with a dirty white fur, thicker along the median line ; mouth moist ; thirst moderate; no appetite; bowels slightly relaxed; and complained of dull pain in the head, back and limbs, with swimming or giddiness in walking; and a great sense of muscular weakness. These symptoms had been gradually developing for three or four days, and clearly indicated the forming stage of typhoid fever, he was directed to go home and keep quiet, take only light food, and for medicine, ten grains of sulphite of soda with eight drops of tincture of gelsemin. every four hours, and a pill containing three grains of quinine and one of blue mass every morning. Two days later a message was left at the office, and Dr. F. H. Davis visited him at his boarding house in Charles street. He found the patient presenting all the ordinary symptoms of typhoid fever, except in relation to the skin, which, instead of being dry, was thoroughly wet with perspiration. Owing to the relaxation of the skin, and some looseness of the bowels, he directed the quinine without the blue mass to be given three times a day, and instead of the sulphite of soda solution, gave an emulsion containing oil of turpentine and tincture of opium. About 48 hours later I saw him in consultation. His skin was still wet with perspiration; his countenance dull, with dryness of the lips and mouth as in ordinary typhoid fever; mind dull, but not wandering; pulse small, weak, and 120 per minute; respiration nearly natural ; urine scanty, and passed with some pain; three or four thin evacuations from the bowels had occurred during the preceding twelve hours; and he was complaining of extreme pain in the region of the lower end of the fibula of the right leg. There was no swelling, no redness, no visible change in the appearance of the leg, except it was paler, or more bloodless, and entirely cold. The coldness was as perfect as though the limb was dead. We could find no pulsation either in the femoral or posterior tibial arteries. All the extremities were cool, and the patient looked very depressed. He was living in a very damp, badly ventilated place, entirely below the level of the street. He was directed powders of sulphate of quinine and morphine, both to relieve the severity of his pains and prevent further intestinal discharges, and dry warmth to the extremities.

In looking at the patient as he lay in his low, damp room, with feeble pulse, cool extremities, relaxed skin, and copious intestinal discharges, the question came up in the mind whether we had a case of pernicious chill, or, as it is more generally termed, congestive intermittant. But the quiet, dull expression of countenance ; the absence of paroxysms of restlessness and tossing; the quiet respiration; and the marked disparity in the temperature of the lower extremities, served to negative the idea of a chill of any kind; and the conviction was forced upon me that some serious mechanical obstruction existed in the arteries supplying the right leg. The cardiac sounds were natural in rhythm, but weak. This fact, with the quiet breathing, led to the conclusion that the obstruction was in the iliac or femoral artery, rather than in the heart or lungs; and as there was no tumor discernable in the abdomen or groin, it was presumed that the obstruction was from emboli or fibrinous clots. The next day the attending physician finding him no better, advised his removal to the hospital, which was done yesterday.

If you examine the present condition of the patient, you still see a dull typhoid expression of countenance; the upper lip is retracted; the exposed parts of the teeth are dry; the tongue coated; the skin dry, but not hot; respiration natural; pulse soft, weak and 115 per minute; abdomen soft; bowels moved three or four times in the twenty-four hours; urine scanty; and both lower extremities cold. The left retains some warmth in the upper part of the thigh, but all below the knee is cold and pale. The right leg is cold throughout, and mottled with purple spots from the foot to the knee, showing commencing gangrene. But there is no swelling and no paralysis, as he moves both limbs at will. This is a very unusual case. A laboring man, in the early period of vigorous manhood, residing in the city only a few months, and boarding in a low, badly ventilated place, is attacked with the ordinary symptoms of typhoid fever, and in four or five days suddenly loses all circulation in the right extremity, which progresses to the development of dry gangrene, and in two or three days more the same state of things takes place in the left. It would seem from the progress of the coldness and arrest of circulation, that complete obstruction occurred first in either the external or common iliac artery of the right side, and gradually extended upward to the abdominal aorta, and to the common iliac of the left side, thus completely interrupting the circulation to both lower extremities.

That the obstruction is in the arterial trunks is evident from the fact that there is no swelling or œdema of the limbs. If the venous trunks were obstructed, the blood would continue to flow into the limbs through the arteries, but not returning through the obstructed veins, its accumulation would necessarily be speedily followed by swelling and more or less serous infiltration into the tissues. You see, however, the reverse of all this in the present case. But what is the nature of the obstruction in the arteries? The absence of all signs of cardiac and pulmonary disease, and also the entire freedom from any abdominal tumors, leaves us only the emboli or fibrinous clots as the probable cause of interrupting the flow of blood to the lower extremities. That the fibrin and perhaps more or less of the albumen of the blood, does sometimes spontaneously solidify forming masses or emboli of greater or less size, which are capable of lodging either in the cavities of the heart or in the blood vessels, and thereby mechanically obstructing the circulation, is a fact familiar to the profession. The exact pathological condition of the blood, which favors or gives rise to such solidification, however, is not well understood. The subject is one that needs an additional amount of careful study, more especially by adding microscopic and chemical analysis to our clinical observations. The latter, so far as my experience goes, appears to show that the formation of emboli occurs chiefly in patients with impaired vital activity or molecular change, with accompanying circumstances such as would favor deficiency of free salts in the blood. It is generally conceded that the albumen, and probably also the fibrine of the blood, is held in solution in the living body by the free alkaline salts, more especially soda and ammonia. Let us see whether these views will aid us in unraveling the pathology of the case before us. The patient is a laboring man who, at work in the heat of summer, had, for two or three weeks previous to being taken down sick, drank profusely of water and more or less of beer and ale. At my first interview with him he acknowledged that he had drank two or three quarts of fluids per day, and had sweat correspondingly profuse. A little reflection will show you how rapidly the composition of the blood must change in some important respects by such a process. All know that *perspiration* contains a notable quantity of saline matter, especially salts of soda; while neither water nor the beer drank supplied these elements. Hence his excessive drinking and consequent excessive perspiration, rapidly exhausted the free alkaline salts, and left these solvents of the albumen and fibrine deficient in the blood. But the evil did not stop here. A normal proportion of saline matter in the blood is necessary to give it the capacity for absorbing and holding the oxygen gas furnished in the air cells of the lungs, or in other words, to render complete the change from venous to arterial blood. This defective arterialization resulted in diminished innervation, muscular weakness, a leaden hue of the lips, a soft weak pulse, and feelings of decided general debility. Add to these, board and lodging in damp, ill-ventilated apartments with the accompanying impurities of a city atmosphere, and you have a fair view of the influences that were at work effecting the important pathological changes from which our patient suffers to-day. The same causes, acting with less intensity, perhaps, affect thousands of the inhabitants of all our cities and populous towns. The changes produced in the blood by the excessive use of drinks and the resulting excessive perspiration, constitute very important pathological conditions, and have more to do with the production of attacks of diarrhœa, cholera morbus, and typhoid fever than is generally supposed. If the circumstances to which we have alluded, or indeed any others, have so far influenced the blood of the patient before us as to give rise to emboli that have completely obstructed the passage of blood to the lower extremities and caused the present appearances of incipient gangrene, it is not likely that any course of treatment will avert a fatal result. It is not likely that any remedy can be safely introduced into the blood in sufficient quantity to re-dissolve a fibrinous clot large enough to block up the iliac arteries. If the deficiencies in the composition of the blood had been corrected by a supply of pure air and a judicious use of the chlorine salts during the preliminary or forming stage of the attack it would probably have prevented both the fever and the arterial obstruction. But in the present state of the patient the prognosis is entirely unfavorable. He should be sustained as much as possible with good nourishment and such medicine as will help sustain the vital properties and nervous force. Carbonate of ammonia and camphor alternated with small doses of strychnine would probably afford the patient as much benefit as anything we could suggest.

The formation of emboli in the cavities of the heart are not very infrequent, and it occasionally happens that the life of a patient laboring under disease of such nature as to cause diminished innervation and cardiac weakness has life terminated suddenly and unexpectedly from this cause.

A few years since I attended a man in feeble general health who was attacked with dysentery. His discharges were largely mixed with blood, but they had continued only about twenty-four hours when he suddenly presented signs of syncope and almost immediately expired. A *post mortem* examination revealed a tenacious white fibrinous clot occupying the right ventricle and extending several inches into the pulmonary artery. It adhered quite closely to the edges of the tricuspid valve as well as to the columnæ corneæ.

Within a few days a neighboring practitioner was attending a case of typhoid dysentery which had presented no unusual symptoms, but was apparently progressing favorably, when there suddenly supervened feelings of great exhaustion, bordering on syncope; the sounds of the heart became muffled and obscure; the pulse extremely feeble, and extremities cold. He passed directly into complete collapse and died in about twelve hours. Although no *post mortem* examination was allowed, yet I have no doubt but a cardiac embolus was the immediate cause of the fatal result.

While speaking of these unusual complications I am reminded of a case recently seen several times in consultation: The patient, a laboring man, aged about twenty-five years, was attacked with a chill, followed by an exceedingly severe pain in his back and loins, with fever of a distinctly remittant type. The urine was scanty and high colored; stomach irritable; the bowels costive, and the pain in the back so severe as to cause the attendant to fear direct inflammation of the lumbar portion of the spinal cord. After a few days of treatment, chiefly with anodynes and alteratives during the febrile exacerbations, quinine and morphine in the remissions, and active revulsives and counter-irritation to the spine, all the more active symptoms subsided. The patient remained quite comfortable for two or three days, though not entirely free from dull pain and soreness both in the lumbar region of the spine and in the direction of the psoas muscles. The tongue remained coated, the urine rather scanty, and a noticeable increase of pain and fever every alternate day, but no chills. The continuance of these symptoms and especially the dull pain and tenderness in the psoas regions led to a suspicion that there might be forming a psoas abscess. But up to the time we now allude to, there had occurred no rigidity or contraction of the psoas muscles causing the thighs to be flexed on the pelvis as is usual in cases involving inflammation or suppuration in the psoas regions. While in this state, about one week after the commencement of his sickness, there came suddenly pain in the left hip, thigh and calf of the leg, accompanied by diffuse swelling of the whole limb. The swelling, tenderness, and pain were greatest on the anterior and outer part of the thigh and in the calf of the leg. There was no redness or erysipelatous appearance of the surface, and but little if any increase of temperatnre. Over the dorsum of the foot and ankle there was sufficient œdema to present pitting on pressure, but the calf of the leg and the whole thigh had a hard or semi-elastic feel, like that of phlegmasia alba dolens. The extent and character of the swelling; the rapidity with which it had been induced, and the preceding pain in the lumbar and psoas regions, led to the belief that some mechanical obstruction existed in the iliac vein, and it was feared that rapid and diffuse suppuration would take place in the cellular tissue of both thigh and leg. The stomach of the patient being irritable and inclined to reject whatever medicine had been given, he was directed nothing but a powder of sub-nitrate of bismuth, six grains, and sulphate of morphia, one-quarter of a grain, every three or four hours, and an emollient poultice of linseed meal over the whole thigh and leg. Under this treatment with mild nourishment he became quite comfortable, the pain gradually declined in the limb, and after two or three days the swelling also began to abate, and in a week it had entirely disappeared without any vestige of suppuration. The patient, though free from fever, remained weak and still suffering from pain and lameness in the small of his back. You will doubtless learn at a future time what becomes of the poor patient before you.*

We might allude to a number of cases presenting unusual complications, but our time will not permit.

Osseous Deposit in Eye.—Mr. Arthur Bracey, of Birmingham (*British Med. Journal*), exhibited at the Pathological and Clinical Section of the Birmingham Branch a specimen of bone found within a disorganized eye which he had excised. The deposit took the form of a cup, and occupied the whole of the inner surface of the choroid. Mr. Bracey stated that authors described similar formations as true bone.—*Med. Record.*

*This patient continued steadily to fail, and died in about one week after he was admitted into the hospital. The right leg had become entirely discolored from gangrene, and the left partially so. A *post mortem* examination revealed no unusual pathological changes in the abdominal or pelvic viscera, except in the abdominal aorta and its branches. Commencing about three-quarters of an inch above the bifurcation, a tough, yellowish white fibrinous clot occupied the vessel and extended through both common iliacs, and on the right side throngh the external iliac to the groin, and the internal iliac two or three inches; also through the external iliac of the right side, but only slightly into the internal iliac. Throughout the extent just mentioned the arteries appeared full, round, and firm, as if injected, while above and below they were empty and collapsed as usual. The coats of the plugged vessels showed no appearance of inflammation.

